# Public Health in British India: A Brief Account of the History of Medical Services and Disease Prevention in Colonial India

**DOI:** 10.4103/0970-0218.45369

**Published:** 2009-01

**Authors:** Muhammad Umair Mushtaq

**Affiliations:** Final Year MBBS Student, President, Student Research Society, Allama Iqbal Medical College, Lahore, Pakistan

The evolution of public health in British India and the history of disease prevention in that part of world in the 19^th^ and early 20^th^ century provides a valuable insight into the period that witnessed the development of new trends in medical systems and a transition from surveys to microscopic studies in medicine. It harbors the earliest laboratory works and groundbreaking achievements in microbiology and immunology. The advent of infectious diseases and tropical medicine was a direct consequence of colonialism. The history of diseases and their prevention in the colonial context traces back the epidemiology of infectious diseases, many of which are still prevalent in third world countries. It reveals the development of surveillance systems and the response to epidemics by the imperial government. It depicts how the establishment of health systems under the colonial power shaped disease control in British India to improve the health of its citizens [Figure 1].

**Figure 1:**
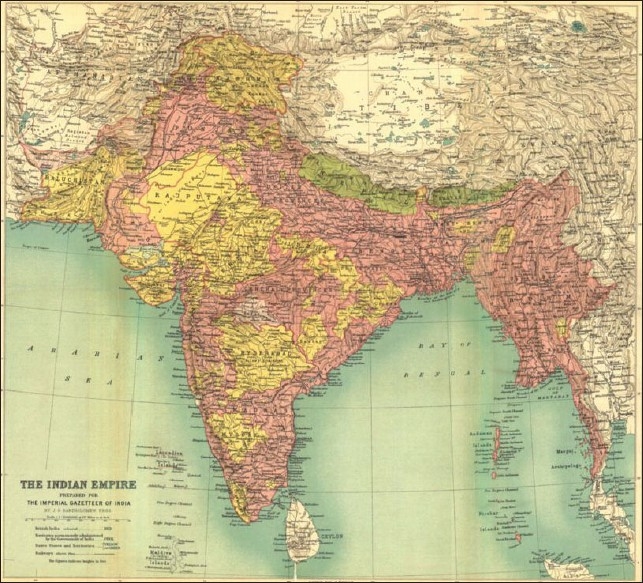
Map of British India

## Establishment of Medical Services

The history of western medicine in India dates back to 1600, when the first medical officers arrived in India along with the British East India Company's first fleet as ship's surgeons. In 1757, the East India Company established its rule in India, which led to the development of civil and military services. A medical department was established in Bengal as far back as 1764, for rendering medical services to the troops and servants of the Company. At that time, it consisted of 4 head surgeons, 8 assistant surgeons, and 28 surgeon's mates. In 1775, Hospital Boards were formed to administer European hospitals comprising of the Surgeon General and Physician General, who were in the staff of the Commander-in-Chief of the Royal Indian Army. In 1785, medical departments were set up in Bengal, Madras, and Bombay presidencies with 234 surgeons. The medical departments involved both military and civil medical services. In 1796, hospital boards were renamed as medical boards to look after the affairs of the civil part of the medical departments. In 1857, the Indian Rebellion led to the transfer of administration of India to the Crown and different departments of civil services were developed. It wasn’t until 1868 that a separate civil medical department was formed in Bengal. In 1869, a Public Health Commissioner and a Statistical Officer were appointed to the Government of India. In 1896, with the abolition of the presidential system, all three presidential medical departments were amalgamated to form the Indian Medical Services (IMS). After the development of IMS, medical duties for the Royal Indian Army were performed by the Army Medical Department, later called the Royal Army Medical Corps (RAMC).(1) Medical departments were under the control of the central government until 1919. The Montgomery-Chelmsford Constitutional Reforms of 1919 led to the transfer of public health, sanitation, and vital statistics to the provinces. This was first step in the decentralization of health administration in India. In 1920-21, Municipality and Local Board Acts were passed containing legal provisions for the advancement of public health in provinces. The Government of India Act 1935 gave further autonomy to provincial governments. All the health activities were categorized in three parts: federal, federal-cum-provincial, and provincial. In 1937, the Central Advisory Board of Health was set up with the Public Health Commissioner as secretary to coordinate the public health activities in the country. In 1939, the Madras Public Health Act was passed, which was the first of its kind in India. In 1946, the Health Survey and Development Committee (Bhore Committee) was appointed by the Government of India to survey the existing health structure in the country and make recommendations for future developments. The Committee submitted its report in 1946 and the health of the nation was reviewed for Public Health, Medical Relief, Professional Education, Medical Research, and International Health.(2,3)

The civil chief medical officer or the person in-charge of the Indian Medical Department was the Director General, Indian Medical Services, who held the rank of Surgeon General. The Director General was under the orders of the Medical Board. He was assisted by the Deputy Director Generals and a team of administrative staff. The Sanitary Commissioner to the government of India supervised sanitation, vaccination, and vital statistics. The Public Health Commissioner and the Statistical Officer were responsible for public health matters. The functions of the central staff were surveys, planning, coordination, programming, and regulation of all health matters in the country. Provincial medical departments were under the control of the local governments of their respective provinces. Principal advisors to the government were the Inspector General of Civil Hospitals (called the Surgeon General in Bombay and Madras), Sanitary Commissioner for the province, and the Director of Public Health. The Deputy Surgeon Generals/Assistant Inspectors assisted the Surgeon General/Inspector General of Civil Hospitals. Provincial officers were responsible for the organization, direction, and inspection of all health facilities. District medical and sanitary arrangements were carried out under the charge of a medical officer called the Civil Surgeon. His duties were to superintend medical institutions and all matters regarding the health of the people. He was required to inspect rural hospitals and dispensaries at least three times a year, perform medico-legal work, and give professional attendance to the superior government officials. He was responsible for sanitary and public health work including vaccinations and vital statistics. The Civil Surgeon was called the District Medical and Sanitary Officer in Madras while in Bombay the Civil Surgeon was only person in charge of the district headquarters. Rural hospitals and dispensaries were under the direct control of the Surgeon General of Bombay. The Deputy Sanitary Commissioners, under the orders of Sanitary Commissioner of Bombay, supervised sanitary work and other public health duties, carried out by the Civil Surgeon in other provinces.(1,4)

The officers of the Indian Medical Services were mostly military surgeons of European origin who were selected in England. In 1788, Lord Cornwallis, Governor General of India, issued orders that medical officers were not permitted to join civil services until serving 2 years in the army and the situation changed little during the rest of British rule.(1,2) In 1835, with the opening of Calcutta Medical College, IMS was opened to the natives of India trained in Calcutta who were selected to serve in Subordinate Military Medical Services or as Assistant Civil Surgeons to serve in sub-divisional civil hospitals. The best of them held minor civil surgeoncies. From 1890 to 1900, ten Indians entered the Indian Medical Services.(1) Later, state medical faculties were established at major provincial headquarters to train technicians who served as Sub-Assistant Civil Surgeons in rural hospitals and dispensaries.

## Medical Institutions

**Figure 2:**
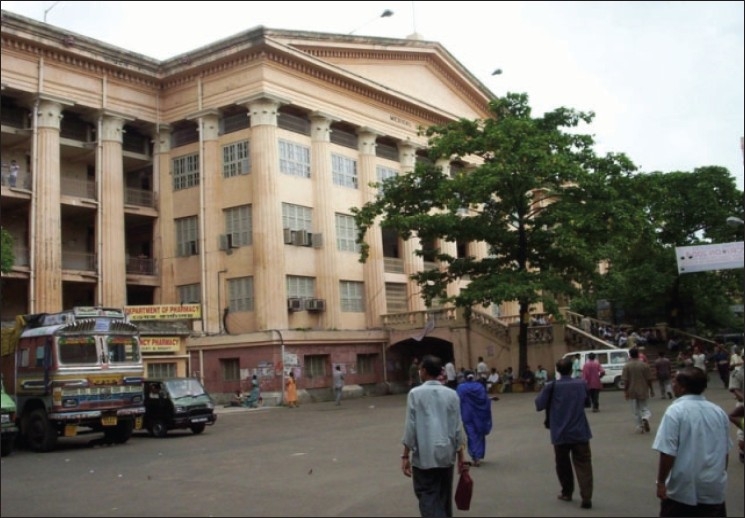
Calcutta Medical College (established in 1835)

**Figure 3:**
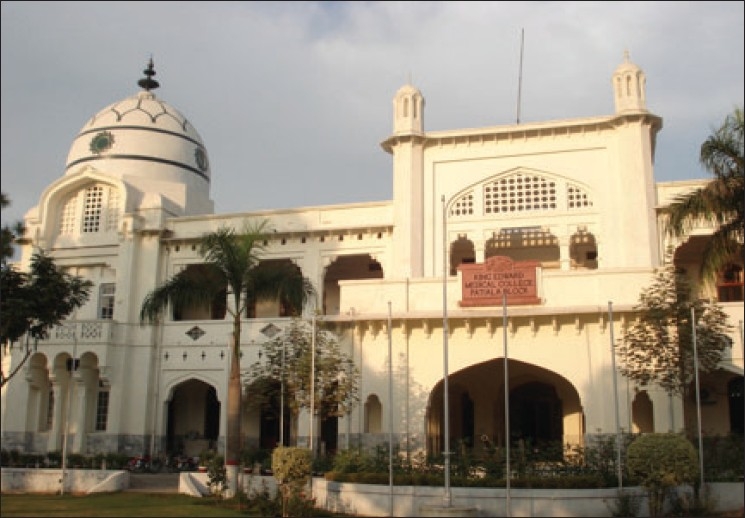
King Edward Medical College, Lahore (established in 1860)

The first hospital in India was the Madras General Hospital in 1679. The Presidency General Hospital, Calcutta was formed in 1796. About four hospitals were formed in Madras between 1800 to1820. To fulfill the growing need for health professionals, Calcutta Medical College was established by an order in February 1835, which was the first institute of western medicine in Asia. Medical College Hospital, Calcutta was formed in 1852 [Figure 2]. In 1860, Lahore Medical School (later named King Edward Medical College) started in Lahore, Punjab [Figure 3]. Afterwards, a network of hospitals was set up throughout India. In 1854, the government of India agreed to supply medicines and instruments to the growing network of minor hospitals and dispensaries. Government Store Depots were established in Calcutta, Madras, Bombay, Mian Mir, and Rangoon.(1) Lady Reading Health School, Dehli was established in 1918. In 1930, the All-India Institute of Hygiene and Public Health was established in Calcutta. In 1939, the first Rural Health Training Center was established in Singur near Calcutta.(3)

The total number of public hospitals and dispensaries under the control of the Imperial government of India was about 1200 in 1880 and in 1902, the figure raised to approximately 2500. There was one hospital for every 330 square miles in 1902. The income of public health facilities was 3.6 million rupees in 1880 and about 8.1 million rupees in 1902. Patient turnover was 7.4 million in 1880; that increased to about 22 million in 1902.(1)

## Mental Health

Lunate asylums were established for insane persons under an act of 1858. These were under the control of the Civil Surgeon of each respective district. From 1895 to 1900, the average number of patients in lunate asylums was about 4600. Central asylums were formed in Bengal, Bombay, Madras, United Provinces, and Punjab. Later, Government Mental Hospitals were established at provincial headquarters. Medical officers were encouraged to attend these facilities and conduct research for improving mental health.(1,4)

## Sanitary Work

The history of sanitary work in British India began with the reports of the Royal Commission of 1859. The commission reported in 1863 on the sanitary conditions of the Army. The mortality rate among British troops was 69 per 1000.(2) The commission recommended the establishment of a Commission of Public Health in each presidency and pointed out the need to improve sanitation and prevention of epidemics in civil society for improving the health of the British Army. Under the Military Cantonments Act of 1864, a sanitary police force was formed under the charge of military medical officers to improve military hygiene. To improve civil sanitary conditions, sanitary boards were formed in each province in 1864. Sanitary Inspector Generals later named as Sanitary Commissioners replaced these boards and took over the charge of sanitation. In 1870, the sanitary department was merged with the vaccination department to form a central sanitary department. From 1870 to 1879, sanitary departments were set up in each province. Under the orders of the Governor General of India in 1880, Sanitary Engineers were employed in all major provinces. The Sanitary Commissioner of India and the provincial sanitary commissioners had no executive powers and were advisors to the government. They carried out the inspection of sanitation, the supervision of vaccinations, the maintenance of vital statistics, and the collection of meteorological data. In districts, civil surgeons (Districts Medical and Sanitary Officers in Madras and Deputy Sanitary Commissioners in Bombay) were in charge of sanitation. The local self-government policies of Lord Ripon strengthened the efforts to improve sanitation by increasing the availability of funds at the local level. In 1885, the Local Self-Government act was passed and local bodies came into existence. These were now responsible for sanitation at the local level but the necessary staff was not provided by the Central Government. In 1912, the Government of India sanctioned the appointment of Deputy Sanitary Commissioners and Health Officers with the local bodies and released funds for sanitation.(1,2,4)

## Vaccination and Vital Statistics

The history of vaccinations can be traced back to 1802 when a Superintendent General of Vaccination was appointed in India after the discovery of the small pox vaccine. In 1827, four European superintendents of vaccination with one Indian vaccinator were appointed to the Bombay presidency. Great efforts were made for vaccination under the charge of the superintendents of vaccination. In 1870, the vaccination work was transferred to the supervision of the Sanitary Commissioners and their staff. The district public vaccination staff was supervised by the Civil Surgeon except in Bombay where it was under the control of the Deputy Sanitary Commissioners. In 1880, an act was passed for the compulsory vaccination of children in municipalities and cantonments. Small pox was the main target during that period, although vaccinations were also carried out for plague and other diseases. Variolation (an Eastern inoculation technique) was also used initially to control small pox. In 1864 and 1865, 556 people were vaccinated in Bengal, the United Provinces, and Punjab while more than 5 million people were vaccinated in the same provinces in 1902 and 1903. In all of British India, the vaccination rate was 2.7% in 1880 and 1881; that number increased to 3.5% in 1902 and 1903. Successful vaccinations at birth were 19.9% in 1880 and 1881 and 39.1% in 1902 and 1903. The budg*et al*located for vaccination was about 0.7 million rupees in 1880 and 1881. That figure rose to approximately 1.1 million rupees in 1902 and 1903.(1,2,4)

In 1873, the Birth and Death Registration act was passed. Vaccination and sanitary staff was responsible for the maintenance of vital statistics i.e., the registration of births and deaths. In 1881, the first Indian Factories Act was passed and the first all-India census was held. To control epidemics, special officers, committees, and commissions were appointed.(3)

## Medico-legal Work and Drugs

At district headquarters, the Civil Surgeons carried out the medico-legal work. At the provincial level, it was under the orders of the Surgeon General Medico-legal. For the purpose of forensic chemical examination and drug testing, laboratories were created at provincial headquarters under the control of the Chief Chemical Examiner. In 1940, the Drugs Act was passed and drugs were made under the control of the government for the first time.(3,4)

## Disease Control and Prevention

When the British Empire came into power in India, they faced the challenge of a new set of diseases that were endemic in that region. India was a vast country with environments ranging from the world's highest mountains to plain green fields, and from tropical forests to barren deserts. Such a diverse region had its own peculiar diseases, which were difficult to prevent with the limited resources of the IMS. Enormous amounts of work was done for the prevention of epidemics to save the lives of people in India in general, and the Imperial troops and officers, in particular. Epidemic diseases that had devastating effects during that period were plague, leprosy, cholera, and malaria. The British government took great efforts to prevent diseases but due to insufficient medical officers and funds, the major target was to alleviate suffering and render curative services as it was solely a state responsibility during that period with virtually no volunteer or private-sector organizations. Prevention and environmental hygiene had long been neglected. It wasn’t until the late 19th century that the government realized that many deaths could be prevented and public health services were strengthened.

## Plague

There are reports of various plague outbreaks in India but trustworthy information is present about the 1812 outbreak in Kutch that spread to Gujarat and Sind, and lasted for approximately 10 years. A disease having all the symptoms of plague was reported in 1828 and 1929 in Hansi in the Hissar district of Punjab. In 1836, plague was reported to be prevalent in the Marwar state of Rajputana. The first official records date back to 1896 when an epidemic of bubonic plague broke out in Bombay. Initially, it was reported in the port cities of Bombay, Pune, Calcutta, and Karachi. In the first year, it was confined to Bombay except for minor occurrences in other parts of the country. In the second year, epidemics were reported in Bengal, Madras, United Provinces, Central Provinces, Punjab, Mysore, Hyderabad, and Kashmir. It devastated almost the whole of India until about 1899. Up to the end of 1903, that deadly epidemic took the lives of about 2 million people according to state records but the actual figures might be much more.(1,5–7)

Being on the international trading route, there was immense pressure on the British Imperial government of India to control this emergency. The Plague Commission was constituted in 1896 under the chairmanship of Prof. T.R. Frasor, Professor of Materia Medica at the University of Edinburgh. It comprised of members from various departments including J.P. Hewett, Interior Secretary to the government of India, Prof. A.E. Wright, Professor of Pathology at Army Medical College Netley, and others. The report of the Plague Commission in 1904 concluded that the disease was highly contagious and considered human transit as an important source of spreading the disease as they carried the germs with them. The commission recommended necessary preventive measures to disinfect and evacuate infected places, to put a control over mass transit, and to improve sanitary conditions. The commission also suggested strengthening of public health services and development of laboratories.(7)

The Epidemic Diseases Act was passed in 1897 and the Governor General of India conferred special powers upon local authorities to implement the necessary measures for control of epidemics. There was a vigorous execution of the act. Colonial power was used for forceful segregation of infected persons, disinfections, evacuation, and even demolition of infected places was carried out. Medical and administrative officials had the right to inspect any suspected person or place; they may have called for detention of any person from ships and railways. That gave rise to many concerns in the native people and riots were reported in some areas but the government used the military power to ensure proper enforcement of necessary preventive measures. Intensive research work was carried out.(8–14) The Plague Research Committee was formed. As indicated in reports of Surgeon Maj. Lyons, President of the Plague Research Committee and by Surgeon Capt. Childe, various types of research was conducted in 1897.(15) The experiments of Hankins concluded that plague bacillus was not spread by saprophytic means from the outside world. Its main sources were poor sanitation and the resultant spread from excretions of humans and animals. Haffkine's Anti-Plague vaccine was used and inoculations were made on a large scale that proved useful as reported by W.B. Bannerman, Superintendent of the Bombay Plague Research Laboratory (1897–1900). Professor Lusting's curative serum was also used and found effective as described by the reports of G. Polverine, Officer in-charge of Parel Municipal Laboratory and Col. J. S. Wilkins Special Medical Officer for Plague Operations.(16)

Detailed surveillance was carried out with individual case histories; camps and field hospitals were established and various extensive reports were drafted. Five plague committees were constituted to monitor the preventive measures. Noteworthy works include Reports on Bubonic Plague Administration in Bombay by M.E. Couchman (1896–1897);(17) Brig. Gen. W.F. Gatacre, Chairman Plague Committee for the year 1896–97;(18) Sir James MacNabb Campbell, Chairman of the Plague Committee for the year 1897–98;(19) the Municipal Commissioner of Bombay for the years 1898 to 1901;(20) and Capt. J.K. Condon for the years 1896–1899.(21) Maj. E. Wilkinson, Chief Plague Medical Officer, also carried out extensive surveys of the epidemic in Punjab as depicted by his reports on plague administration and inoculation in the plague infected areas of Punjab and its dependencies (1901–1903).(22–24)

## Leprosy

Leprosy was a big problem in British India. IMS medical officers did enormous amounts of research on the scientific treatment for leprosy. Despite its limitations and hardships, leprosy research in India received worldwide recognition; many Indian remedies for leprosy have been incorporated into western medicine.

Because of G.A. Hansen's discovery in 1873 that leprosy is spread by contact, H.V. Carter of the Bengal Medical Department gained an authority over leprosy control in India. He earned great recognition in the central imperial government of India and suggested isolating lepers. He urged the establishment of Leper Asylums in India as these were formed in Norway in those days.(25,26) After the 1889 Leprosy Bill, the National Leprosy Fund was constituted by the British Empire under chairmanship of the Prince of Wales. A Leprosy Commission was formed to investigate the etiology and epidemiology of leprosy. The Leprosy Commission concluded that leprosy is a disease *sui generis* caused by a bacillus having striking resemblance to tuberculosis. It is not a hereditary disease, there is spread by contagious means but the chances for that are very small. However, its spread is indirectly influenced by poor sanitation and malnutrition. The Commission suggested that segregation might not be fruitful in India. It suggested a prohibition on the sale of food articles, prostitution, and other occupations involving direct interference with people like barbers or watermen by the infected people. It insisted on the improvement of sanitary and living conditions.(27) However, the government of India passed the All-India Leprosy Act in 1898 and Leper Asylums were established in major parts of the country and forcible segregation of lepers was carried out.

Different provinces furnished various surveys and reports. Excessive surveillance and research work was carried out on the distribution of lepers, hereditary transmission, and predisposition possibilities, contagiousness, and relation of disease with sanitation and diet.(28–30) In 1881, there were approximately 120,000 patients with leprosy in India that decreased to 102,000 in 1921. These were excellent statistics keeping in view the high birth rate in India.(1)

## Cholera

Officers of the British East India Company were not familiar with cholera. Before 1817, cholera was confined to Bengal but the 1817–1821 cholera epidemics in India shocked the Company. By the 1830s, cholera was known to be a life-threatening disease to the western world. In India, it gained the focus of medical services due to its serious impact on the troops and officers of the Company; otherwise, it was a disease of poor people. Due to the lack of effective treatment for cholera in that period, the main focus was set on its prevention.(1,5)

The British Indian government stuck to metrological theories about cholera after the Constantinople International Sanitary Conference of 1868, believing that atmospheric conditions are the basic cause of spreading the disease. After the 1868 cholera epidemic in India, the Cholera Committee was set up to investigate the causes of the disease. It comprised of the Principal Inspector General of the Indian Medical Department, Sanitary Commissioner for Madras, and Col. A.C. Silver. The origin and generation of cholera, the epidemicity and endemicity of the disease in India, transmissibility and propagation of cholera, and measures necessary for its prevention were studied. The committee concluded that cholera was frequent especially at religious festivals and fairs. Epidemics were attributable to the importation of disease by pilgrims, travelers, and troops. The committee suggested improving sanitation, ensuring proper management of festivals, and developing hygienic conditions in institutions like hospitals, jails, and military cantonments.(31,32) Dr. S.C. Townsend, Sanitary Commissioner for Central Provinces and Berar, also reported his 1868 cholera epidemic investigations.(33)

In the 1860s and 1870s, Dr. James L. Bryden, India's first epidemiologist and government's chief advisor on epidemic cholera, studied cholera extensively. He had first-hand experience with cholera during his work as a statistical officer in IMS in Bengal. But, he considered cholera to be an air-borne disease probably spread by a seed-like organism. He reported that cholera is not transmitted by contaminated water. A.C. DeRenzy, Sanitary Commissioner of Punjab, opposed his views and stated that it would hurt the sanitary work going on in India to prevent the spread of cholera.(2)

John Murray, who served as Inspector General of Civil Hospitals in North Western Provinces and Bengal, conducted detailed studies on cholera. Although there was evidence of contagiousness, Murray believed that environmental factors precipitated the attacks of cholera, but he gave valuable treatment guidelines for cholera in that period.(34) Surgeon W.R. Cornish, Sanitary Commissioner for Madras, challenged metrological theories about the spread of cholera and carried out detailed surveillance and research work to establish the contagiousness of disease, which is evident from his reports on cholera in Southern India in 1871 and his investigations of cholera outbreaks in H.M.'s 18^th^ Hussars in Secunderabad in 1871 that killed 115 people in 1 month including 20 troops of the Royal Indian Army.(35)

Other significant works include reports from H.W. Bellew, Deputy Surgeon General and Sanitary Commissioner for Punjab, about the cholera outbreaks in India from 1862 to 1881, and the reports of Commissioner Benarus Division (United Provinces) about the disease outbreaks in the sub-division of Bulliah and the district of Mirzapore.(36) In the 1890s, metrological theories about cholera were abandoned, as desired by W.R. Cornish. New treatment options evolved along with better prevention methods resulting in the marked decrease in cholera mortality.

## Malaria

Fever was one of the leading causes of deaths in India. The situation worsened in the early 19^th^ century. One of the contributing factors was the establishment of the railways and irrigation network by the British government of India without keeping in view the efficient drainage systems for floods and rainwaters. This created many fresh water reservoirs for the propagation of mosquitoes. Due to the heavy death toll, economic loss, and risk to the lives of British officers serving in vulnerable areas like Punjab, a lot of research was done for malaria control. In the 1840s, attention was paid to proper drainage and chemoprophylaxis was started with Quinine.(1,5)

**Figure 4:**
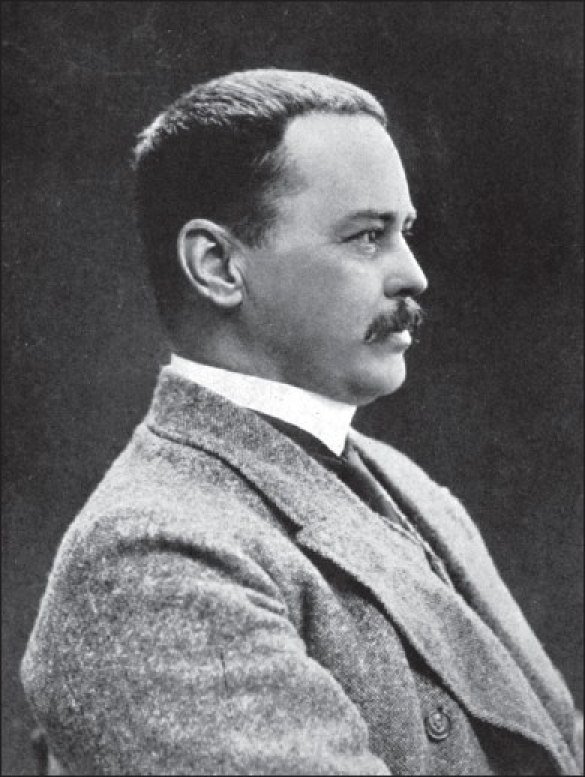
Surgeon Major Sir Ronald Ross (1857-1932)

Surgeon Major Sir Ronald Ross joined the Indian Medical Services in 1881 [Figure 4]. He started to study malaria in 1882. Despite initial failures and hardships, his devotion to research for nearly 2 1/2 years earned him great honor. In August 1897, he demonstrated the life cycle of the malarial parasite stating that anopheles mosquitoes carried the protozoan parasites called “plasmodia”.(37) He was later knighted and given a Nobel Prize in Medicine in 1902. This discovery opened new horizons in malaria research and shaped the malaria control programs toward a new direction mainly focusing on the eradication of mosquitoes.

In 1900, Christophers, Stephens, and James conducted detailed research on mosquitoes in the military cantonments in Punjab. All-India Malaria Conferences (1900–1909) and Punjab Malaria Surveys (1909–1911) were carried out under the supervision of Christophers.(5) From 1903 to 1908, Capt. S.P. James conducted research on the causation and prevention of malarial fevers. He wrote useful reports for the prevention and treatment of malaria for health care providers.(38,39) In 1909, the Central Malaria Bureau was formed in Kasauli for malaria control and investigations.(3,5)

Capt. S.R. Christophers and Dr. C.A. Bentley investigated the malaria and black-water fever in Duars in 1911. They reported that it was an area of malaria hyper-endemicity with an endemic index of 50–100% in the district. Black water fever was one of the consequences of hyper-endemic malaria. Large-scale tropical aggregation of labor had an important role in the epidemiology of malaria in the tropics. They found that a lack of registration of vital occurrences; malnutrition, differential labor system, poor sanitation, and the formation of foci with infected immigrants were responsible for epidemicity in that region.(40) On the special orders of the Malaria Investigation Committee, Charles A. Bentley studied causes and prevention of malaria in Bombay in 1911. His reports revealed that 60–75% of all fever cases were due to malaria and these resulted in the economic loss of not less than 1.2 million rupees. Bently suggested efficient mosquito eradication and the improvement of drainage systems for malaria control.(41) In 1913, Maj. J.L. Marjoribanks, Deputy Sanitary Commissioner for Western Registration Districts, studied malaria in the Islands of Salsette and drew similar conclusions.(42)

Malaria was a major problem in Punjab. After initial works by Christophers, the Punjab Malaria Bureau carried out detailed surveillance and research on malaria. It is evident from the extensive investigations and reports of Chief Malaria Medical Officers of Punjab from 1913 to 1918 who were Capt. Clifford A. Gill (1913), Lt. Col. D.T. Lane (1914), Col. H. Hendley (1915-1917), and Col. R.C. MacWatt (1918), that malaria was a major problem in Punjab and extensive work was done for its prevention and control. These volumes are the comprehensive summary of works on malaria control in Punjab in those 6 years. The malarial death rate was 17.15/1000 in 1913, 27.613/1000 in 1915, 14.73/1000 in 1916, and 66.56/million in 1918.(43–48)

Research on malarial vectors i.e., mosquitoes were also performed. After Stephens and Christophers in 1900, noteworthy works on mosquitoes were the Stegomyia survey by Maj. McGilchrist in 1912–1913, the Survey of Malaria and Environs in Calcutta by M.O.T. Iyengar, Entomologist to the Department of Malaria Research of Bengal, and the Mosquito Survey in Karachi by Dr. K.S. Mhaskar in 1913.(5,49,50) The League of Nations criticized the chemoprophylaxis with Quinine that was practiced on a large scale by the British Indian government. In high-risk areas like some parts of Punjab and the tropics, quinine was made available at special institutions like jails and post offices in small packs that contained 5–7 quinine granules with a price of only quarter-anna. However, it remained so until the introduction of chloroquine and WHO guidelines for the use of DDT.(1,5)

## Other Communicable Diseases

In the province of Assam, Indian Officers faced a strange disease endemic called Kala-azar and Beriberi by the natives. An investigation about Kala-azar was carried out by G.M. Giles, Surgeon IMS on special duty in Assam in 1898. He concluded that the disease was anchylostomiasis with slightly different symptoms.(51) In 1899, Surgeon Major Ronald Ross investigated the disease and reported that Kala-azar was an epidemic and communicable disease with symptoms resembling those of malaria except hepatomegaly and spleenomegaly. Spleen and liver enlargement observed in these cases by Ross was not a character of malaria. Anchylostomas were found but these do not cause such symptoms. Ross concluded that it was not a malarial fever but a disease microscopically and macroscopically similar, except for the absence of parasites and melanin; and the presence of visceral invasion especially that of spleen and liver.(52) This initial research provided a base for future researchers and the disease was later proved to be visceral leishmaniasis caused by a protozoan Leishmania donovani.

T.G. Hewlett studied enteric fever in 1883 and conducted detailed studies on individual case histories and environmental conditions.(53) The Sleeping Sickness commission (1908–1910) was formed to investigate the causes of the disease. Capt. F.P. Mackie studied the disease and preventive measures.(54) Other significant works were on yellow fever by S.P. James in 1913 and on Hookworm disease by Maj. Clayton Lane in 1914.(55,56)

Tuberculosis had long been recognized as a lethal disease. It was present in India especially in lower socio-economic classes. In 1939, The Tuberculosis Foundation of India was established. As there was no clinically effective treatment available for tuberculosis at that time, tuberculosis sanatoriums were formed in hilly areas to provide a healthy environment and segregation.(1,3)

## Research

Although there were groundbreaking works on a variety of diseases, which proved to be very helpful in the prevention of epidemics, the British government of India discouraged innovation and research due to a lack of funds and other difficulties. This branch of medical systems had long been neglected in India. In the late 19^th^ and early 20^th^ century, situations improved and it was widely accepted that medical research was an integral part of preventive medicine. In 1884, the foundation stone of India's first medical laboratory was laid down. A central laboratory was established in Kasauli near Simla.(1) It was for research purposes and to act as a reference public health laboratory. Provincial laboratories were then established at major provincial headquarters to carry out public health and bacteriological laboratory work. In 1900, the Indian Pasteur Institute for the treatment of patients bitten by rabid animals was formed in Kasauli and later such institutes were also formed in other parts of the country.(1) In 1911, the Indian Research Fund Association was formed to provide funds for research projects. A Nutritional Research Laboratory was set up in Coonoor in 1918.(3)

The British Imperial government set up and strengthened an organized medical system in Colonial India that replaced the indigenous Indian and Arabic medicine systems. Slow progress in early years was due to indifference on the part of people and a lack of funds and medical professionals on the part of the government. The people of India resisted the British colonialism, and they were reluctant to support any services by the foreign government. These trends slowly changed as the natives were educated according to the British system. They then decided to serve in Indian civil and military services and lessen their hardships by taking part in government affairs. That is why Indian Medical Services flourished in the late 19^th^ and early 20^th^ century. There were dramatic improvements in medical and sanitary conditions in British India. IMS efficiently coped up with deadly epidemics like the plague and cholera. Almost all the diseases prevalent at that time in India like small pox, leprosy, and malaria were controlled successfully. There were very few epidemics in later years and many of the diseases were almost eradicated. Officers and researchers of Indian Medical Services contributed a lot to the study and prevention of diseases. The role of medical officers serving in India should be better judged by their aspirations, priorities, and limitations. Although the archetypical colonial design of medical services, Eurocentric policies, and neglect of the indigenous population failed to relieve the plight of the poor for many years, the work completed during that period of time formed the basis of what we have achieved today to improve the health of people.
